# HnRNP L is essential for peripheral T cell proliferation and survival

**DOI:** 10.3389/fimmu.2025.1543145

**Published:** 2025-04-10

**Authors:** Tristan L. A. White, Devin J. Henry, Sean D. A. Roberts, Ye Jin, Yi-Nan Gong, Penelope A. Morel

**Affiliations:** Department of Immunology, University of Pittsburgh School of Medicine, Pittsburgh, PA, United States

**Keywords:** T cell activation, proliferation, RNA binding proteins, T helper cell subsets, apoptosis, hnRNP L

## Abstract

**Introduction:**

During T cell development, heterogeneous nuclear ribonucleoprotein (hnRNP) L is known to regulate CD4 T helper subset differentiation, the proliferation and migration of thymocytes, as loss of hnRNP L in early T cell development results in a failure of T cells to reach the periphery.

**Methods:**

To better understand the role of hnRNP L in modulating peripheral T cell trafficking and function, we analyzed T survival and activation in newly generated CD4Cre x hnRNP L^Fl/Fl^ (KO) mice. *In vitro* and *in vivo* analyses of CD4 T cell differentiation, T cell proliferation and death post activation were performed.

**Results:**

Our initial study of the steady state profile of the KO mice showed normal migration of T cells from the thymus, but peripheral T cell numbers were reduced. Analysis of TCR-mediated signaling pathways revealed normal early T cell activation. However, T cells lacking hnRNP L had marked defects in their ability to differentiate into T helper cell subsets due to reduced proliferation and increased death. In vivo, using immunization studies, KO CD4 T cells failed to fully differentiate into T follicular helper (Tfh) cells and were unable to support the formation of germinal center B cells. Death of activated hnRNP L KO cells could be reversed by treating the cells with zVAD, a pan-caspase inhibitor. In addition, hnRNP L KO cells failed to upregulate the anti-apoptotic protein Bcl-XL following activation.

**Discussion:**

These studies suggest that hnRNP L plays an important role in T cell activation and survival. Our studies suggest that hnRNP L plays a critical pro-survival role in activated T cells and that alternative splicing of factors that prevent apoptosis may be an important mechanism by which this is achieved.

## Introduction

We have previously shown that the strength of the initial TCR signal influences the differentiation of specific CD4^+^ T cell subsets. Notably, TCR signal strength has a major impact on the activation of the PI3K/Akt/mTOR pathway, which influences T helper (Th) cell fate (1–4). Stimulating naïve T cells with a low dose of a high-affinity antigen (Ag) fails to fully activate the PI3K/Akt/mTOR signaling pathway (2), favors Treg fate (1, 5), and Th2 cell differentiation (6–8) while high Ag dose stimulation fully activates the PI3K/Akt/mTOR pathway leading to inflammatory Th1 cell differentiation (1, 2, 4, 7). PI3K/Akt/mTOR signaling strength determines the Akt phosphorylation state and substrate specificity. When Akt is fully activated following high-dose stimulation, Akt is phosphorylated at two sites: Ser473 and Thr308 (9). In this state, Akt phosphorylates substrates such as FoxO1 (3), which inhibits Treg induction (1, 3–10). Under low-dose Ag stimulation, Akt is only phosphorylated at Thr308 and the lack of phosphorylation at Ser473 results from a reduction in mTORC2 activity (11). We have shown that Akt phosphorylates different sets of substrates under these two activation conditions, and we observed the phosphorylation of hnRNP L and other RBPs in Treg-inducing conditions (3). Furthermore, siRNA knockdown of hnRNP L inhibited the generation of Treg induced under low-dose conditions (3).

HnRNP L functions in many biological processes, such as DNA repair, RNA alternative splicing, transcription factor activity, protein translation, cell signal transduction, and gene expression (12). One of hnRNP L’s most notable functions is alternative splicing (13), and it is known to play a role in the splicing of CD45 (14). HnRNP L binds to specific RNA sequences within pre-mRNA transcripts and influences splice site selection, exon inclusion/exclusion, and alternative exon usage (15). HnRNP L promotes the stabilization of target mRNAs by protecting the mRNAs from degradation. HnRNP L can influence translation efficiency controlled by IRES within mRNA molecules (16).

Loss of hnRNP L in thymocytes has several effects on cell development. In the Lck^cre^ x *Hnrnpl^fl/fl^* mouse model, hnRNP L-deficient T cells failed to move beyond the DN stage and were unable to migrate from the thymus to the periphery (17). The few cells that could be found in the periphery were cells that failed to delete hnRNP L. In a transcriptome-wide RNA profiling study, hnRNP L was found to bind to 41 transcripts in the Wnt signaling pathway and 38 transcripts in the TCR signaling pathway (18). In the Lck^cre^ x *Hnrnpl^fl/fl^* mouse model, hnRNP L-deficient DN4 thymocytes at steady state and upon anti-CD3 activation had lower levels of Lck phosphorylation at tyrosine 505 (17). HnRNP L regulates the alternative splicing of exon 4 in CD45 mRNA upon T cell activation (17, 19).

Earlier studies demonstrated the role of hnRNP L in maintaining the survival of hematopoietic stem cells (HSC) (20). Using the Vav-Cre x *Hnrnpl^fl/fl^* mouse model, the investigators demonstrated severe defects in global hematopoiesis and showed that hnRNP L was necessary for HSC self-renewal. The absence of hnRNP L at this stage led to apoptosis of HSCs, and this was associated with the activation of various death receptors (20) In addition, the cells could be rescued by blocking the apoptotic pathway (20).

Our previous studies suggested a role for hnRNP L in Th cell subset differentiation (3) and thus, we were interested to explore the role of this RNA binding protein at later stages of T cell development. In these studies, we used a CD4^cre^ x *Hnrnpl^fl/fl^* mouse model, which depletes hnRNP L at the DP stage of T cell development, and we studied the effects of hnRNP L depletion on peripheral CD4**^+^** T cell fate and function. This mouse model demonstrated normal thymic development, and thus, we were able to assess the role of hnRNP L on peripheral T cell survival, activation, and differentiation both *in vitro* and *in vivo.* These studies demonstrate that hnRNP L is essential for peripheral T cell activation and survival. While early activation signals appeared intact, hnRNP L KO T cells failed to differentiate into Th cell subsets, *in vitro* and *in vivo*, and were observed to undergo apoptosis. This appeared to be associated with a failure to upregulate the anti-apoptotic protein BCL-X_L._

## Materials and methods

### Mice

The *hnrnpl^fl/fl^* mice were a kind gift from Dr. Tarik Möröy, Université de Montréal, Canada. B6.Cg-Tg(Cd4-cre)1Cwi/BfluJ and C57BL/6 mice were purchased from The Jackson Laboratory. Mice from the *hnrnpl^fl/fl^* and the CD4-cre lines were bred together to generate CD4cre positive x *hnrnpl^fl/fl^* homozygotes (KO) and F1 mice were then bred to *hnrnpl^fl/fl^* (WT). This cross resulted in the generation of KO, and litter mate control WT mice. All mice were age and sex-matched for experiments. All mice were housed in a specific pathogen-free facility at the University of Pittsburgh and were treated under Institutional Animal Care and Use Committee-approved guidelines in accordance with approved protocol.

### Immuno-profiling, cell staining, and flow cytometry

Spleen, thymus, and lymph nodes (axillary, brachial, and inguinal were pooled) were isolated from mice ages 6-8 weeks. Single cell suspensions were created by passing the spleen through a 40μm cell strainer into a 50 ml Falcon tube, followed by washing the strainer with cold PBS. Red blood cells from the spleen cell suspensions were lysed in ACK lysing buffer (Thermo Fisher) for 2 min, then washed in FACS buffer.

Single-cell suspensions were stained with Abs for: TCRß-BV605 (H57-597; BD Biosciences), CD4-BUV395 (GK1.5; BD Biosciences), CD8a -PE-Cy5 (53-6.7; Thermo Fisher), CD62-BV650 (MEL-14; BD Biosciences, CD25-Alexa Fluor 700 (PC61.5;BioLegend), CD44-BV510 (IM7; BD Biosciences), GARP-PE-Cy7 (F011-5; BioLegend), NRP-1-PE (3D5304M; BioLegend), Helios-APC eFlour780 (22F6; Thermo Fisher), Foxp3-Alexa Fluor 450 (FJK-19S; Thermo Fisher), T-BET-Percp-Cy5.5 (4B10; Thermo Fisher), GATA3-PE (L50-823; BD Biosciences), RORγT-APC (AFKJS-9; Thermo Fisher), B220-FITC (RA3-6B2;Thermo Fisher), CD138-BV605 (281-2; BD Biosciences), CD27-APC (LG.7F9; Thermo Fisher), CD19-BV480 (1D3; BD Biosciences), CD93-PE (AA4.1; Thermo Fisher), ICOS-FITC (DX29; BioLegend), CXCR5-PE (RF8B2; BD Biosciences), CD38- PerCP-Cy5.5 (HIT2; BD Bioscience), CD95-PE-Cy7(DX2; Thermo Fisher), NP-APC, BCL6-PeCy7 (K112-91; BD Bioscience) HnRNPL- Biotin (4D11:NOVUS); Strep-APC (Biolegend). Dead cells were discriminated by staining with Zombie NIR Dye (BD Bioscience) with surface staining on ice for 30 min in PBS. For intracellular staining of transcription factors, cells were stained with surface markers, fixed in Fix/Perm buffer (eBioscience) overnight, washed twice in permeabilization buffer (eBioscience), and stained in permeabilization buffer for 2 hours on ice. Samples were acquired on the Aurora (Cytek) and analyzed by FlowJo (Tree Star). For the identification of various immune cell populations, we first sub-gated on live single cells, and subsequent gates are indicated in the figure legends.

### CFSE cell staining

T cells were resuspended in plain PBS at 1x10^6^/mL with a minimal volume of 0.5ml. Separately CFSE was diluted to 1:500 in PBS. T cells were vortexed gently with CFSE diluent at a final concentration 1:1000. T cells were incubated at 37°C for 15 mins. Prewarmed media was added and the cells were washed two times in media.

### *In vitro* skewing assays

Splenic T cells were isolated using a pan T cell isolation kit (Miltenyi Biotec; Cat # 130-095-130) from WT and KO mice. 96 well U-bottomed plates (Falcon) were coated with anti-CD3 (17A2; Thermo Fisher) at various doses for 2hr at 37°C. In cytokine skews 2-2.5x10^5,^ T cells were stimulated under the following conditions: All cells were activated with 1.0 µg/ml of plate-bound anti-CD3 and 1.0 µg/ml of soluble anti-CD28 (37.51; Thermo Fisher) and the indicated cytokine cocktails; Th0 (10 µg/ml of anti-IFNγ, 10 µg/ml of anti-IL-4 and, 20 U/ml of IL-2); Th1 (10 ng/ml of IL-12, 10µg/ml of anti-IL-4 and, 20 U/ml of IL-2); Th2 (10 ng/mL of IL-4, 10 µg/ml of anti-IFNγ and, 20 U/ml of IL-2); Th17 (2.5 ng/ml of TGF-β, 20 ng/ml of IL-6, 10 µg/mL of anti-IFNγ, 10 µg/ml of anti-IL4 and, 20 U/ml of anti-IL-2); iTregs (5 ng/ml of TGF-β, 100 U/ml of IL-2, 10 µg/ml of anti-IFNγ and, 10 µg/ml of anti-IL4). The cells were harvested after 3 days.

### NP immunization

Mice 8-12 weeks of age were immunized intraperitoneally with 100 µg NP-keyhole limpet hemocyanin (NP-KLH) precipitated in Alum (21). On day 10 post-immunization, the spleens were isolated and assessed via flow cytometry for the presence of Tfh cells and NP-specific B cells and germinal center B cells.

### T-cell activation assay

T cells from WT and KO mice were isolated using a pan T cell isolation kit (Miltenyi Biotec; Cat # 130-095-130). 2-2.5x10^5^ T cells were plated in a 96-well plate with plate-bound anti-CD3 (3.0 µg/ml) in the presence of soluble anti-CD28 (3.0 µg/ml). The cells were harvested at various time points and analyzed for signaling and activation markers by flow cytometry.

### Death assay

T cells from WT and KO mice were isolated using a pan T cell isolation kit (Miltenyi Biotec; Cat # 130-095-130). 2-2.5x10^5^ T cells were plated in a 96-well plate (Falcon) with various cell death pathway inhibitors for 1hr, including zVAD-FMK (1000X), ZHAR-99 (500X), Lip1 (500 X), Fer-1 (500X) and DMSO. Then, the cells were activated with plate-bound anti-CD3 (3.0 µg/ml) in the presence of soluble anti-CD28 (3.0 µg/ml). The cells were harvested at various time points and analyzed for survival by flow cytometry.

### Western blot

Naïve CD4^+^ cells were isolated using a naïve CD4^+^ T cell isolation kit (Miltenyi Biotec, cat# 130-104-453) from WT (*hnrnpl^fl/fl^)* and KO mice. 2-2.5x10^5^ CD4^+^ T cells were activated with plate-bound anti-CD3 (3.0 µg/ml) in the presence of soluble anti-CD28 (3.0 µg/ml) for various times. Cells were lysed in ice-cold lysis buffer (Cell Signaling) with protease inhibitor (Thermo Fisher) on ice for 40 min. Western blot was performed by first blotting for BCL-X_L_ (54H6: Cell signaling), then blotting for hnRNP L (4D11: NOVIS), and finally Beta-Actin (13E5: Cell signaling).

### Quantification and statistical analysis

Data are presented as mean ± SD, including n for each experiment, representing the number of mice used per group unless otherwise stated. Statistical significance was determined using unpaired Student’s t-test when comparing two groups and one-way or two way ANOVA with Tukey’s multiple comparisons posttest when comparing more than two groups. All statistical analysis was calculated using Prism software (GraphPad).

## Results

### T cell characterization of the CD4^cre^ x *Hnrnpl^fl/fl^* mouse model

**Figure 1:**
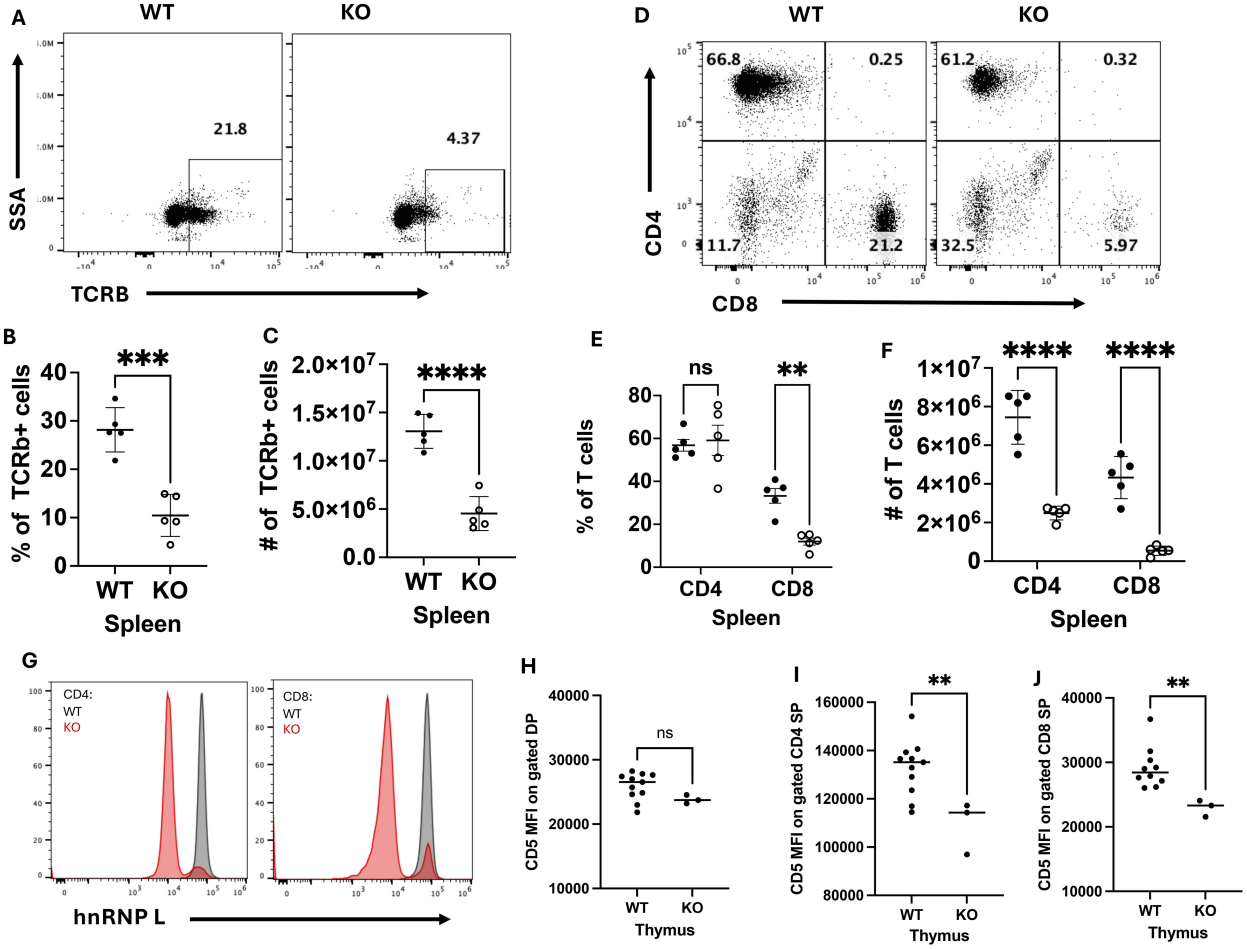
HnRNP L deficient mice have a reduction in peripheral T cells. Splenocytes were isolated from WT and KO mice to assess T cell number and phenotype. A representative flow plot of **(A)** TCRβ^+^ cells and of **(B)** CD4^+^ and CD8^+^ T cells in the spleen of WT and KO mice. The frequency of **(C)** and number **(D)** of TCRβ^+^ cells in the spleen, and the frequency of **(E)** and number **(F)** of CD4^+^ and CD8^+^ T cells in the spleen. **(G)** The representative flow plot of hnRNP L expression in CD4^+^ and CD8^+^ T cells. Thymocytes were assessed for the expression of CD5 in DP, CD4 and CD8 SP thymocytes. Geometric MFI of CD5 in **(H)** DP, **(I)** CD4 SP and **(J)** CD8 thymocytes SP. Data is representative of five independent experiments for **(A-G)**, and the combination of two independent experiments **(H-J)**. A two-way ANOVA analysis with Tukey post-test was performed **(E, F)** and an Unpaired T-Test was performed **(C, D, H-J)**, **p < 0.01, ***p < 0.001, ****p<0.0001. ns, not significant.

To determine whether loss of hnRNP L at the DP stage of T cell development had an impact on T cell development, we profiled T cell development in the thymus by flow cytometry (**Supplementary Figure 1A**). There were no significant differences in the percentage (**Supplementary Figure 1B**) or number (**Supplementary Figure 1C**) of double negative (DN), double positive (DP), CD4^+^ single positive (SP), and CD8^+^ SP thymocytes. T cells were isolated from the spleen and lymph nodes (LN) of WT and KO mice to assess T cell populations in the periphery. HnRNP L deficient mice had significant reductions in the percentage (**Figures 1A, B**) and number of TCRβ^+^ cells (**Figure 1C**) in the spleen. In the LN, there was a decrease in the number of TCRβ^+^ cells (**Supplemenatry Figure 1E**) but not the frequency of TCRβ^+^ cells (**Supplementary Figure 1D**). Within the T cell population, there was no significant difference in the frequency of CD4^+^ T cells, but there was a decrease in the frequency of CD8^+^ T cells in the spleen (**Figures 1D, E**) and in the LNs there was no significant difference in the percentage of CD4^+^ or CD8^+^ T cells (**Supplementary Figure 1F**). There was a significant decrease in the number of CD4^+^ T and CD8^+^ T cells in the spleen (**Figure 1F**) and LNs (**Supplementary Figure 1G**). Further analysis revealed no changes in the number or percentages of naïve CD4^+^ and CD8^+^ T cells or in the number or percentages of FoxP3^+^ Treg (data not shown). Staining for the expression of hnRNP L in peripheral T cells confirmed that the vast majority of CD4^+^ T and CD8^+^ T cells had deleted hnRNP L (**Figure 1G**), suggesting that there was no defect in the migration of mature T cells from the thymus to the periphery. Due to the dramatic decrease in peripheral T cell numbers, we wondered if there were any changes in thymic selection or survival of T cells in the periphery. The level of CD5 expression has been reported to correlate with TCR signal strength in thymocytes undergoing selection (22, 23) Thus, we examined CD5 expression levels in DP, CD4 SP and CD8 SP thymocytes in WT and KO animals. No significant differences in CD5 gMFI were observed in DP thymocytes (**Figure 1H**) but the expression level of CD5 was significantly lower in KO CD4 (**Figure 1I**) and CD8 (**Figure 1J**) SP thymocytes compared to WT. These data suggested that KO CD4^+^ and CD8^+^ cells surviving in the periphery had experienced weaker TCR signals than their WT counterparts.

### Early pS6 and CD69 expression is not affected in hnRNP L-deficient T cells

**Figure 2:**
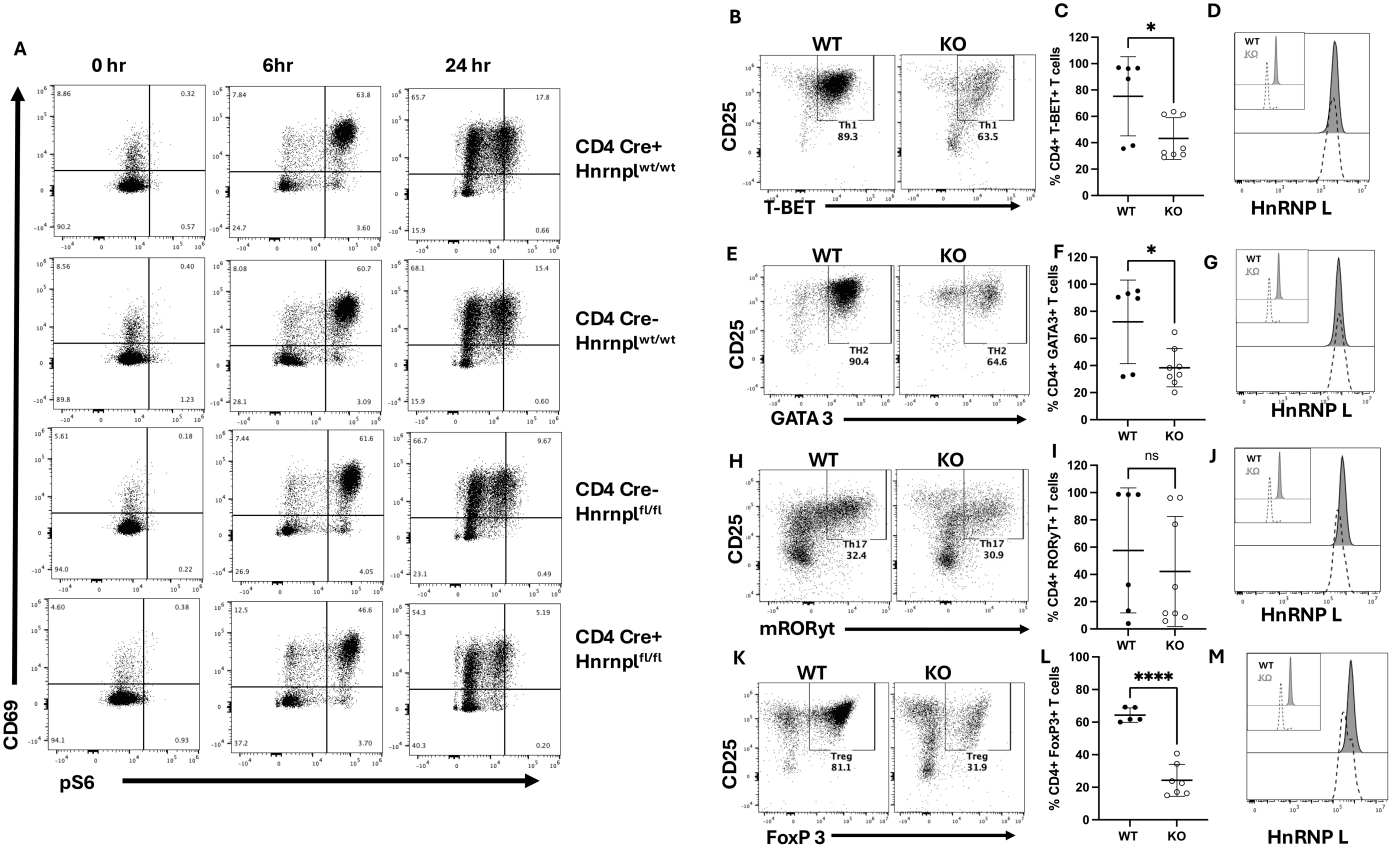
Early pS6, and CD69, and CD25 expression are not affected in hnRNP L-deficient T cells, but hnRNP L is required for Th1, Th2, Th17, and Treg differentiation. T cells from WT (CD4Cre^+^ hnrnpl^wt/wt^, CD4Cre^-^ hnrnpl^wt/wt^, CD4Cre^-^ hnrnpfl^fl/fl^) and KO (CD4Cre^+^ hnrnpfl^fl/fl^) mice were isolated using a negative selection pan T cell isolation kit. 2-2.5x10^5^ T cells were plated in a 96-well plate with 3.0 µg/ml plate-bound anti-CD3 in the presence of soluble 3.0 µg/ml anti-CD28. The cells were harvested after various time points. **(A)** Representative flow plot of stimulated CD4^+^ T cell expression of CD69 vs pS6 at the indicated time points. T cells from WT and KO mice were stimulated with IL-12, anti-IL-4, and IL-2 to induce Th1 cells. **(B)** Representative flow plot of T-Bet^+^ CD25^+^ T cells three days post stimulation, gated on CD4^+^ T cells, **(C)** the percentage of Tbet^+^ CD4^+^ T cells over several experiments, and **(D)**, a histogram of hnRNP L expression in WT (filled histogram) and KO (dotted line) Tbet^+^ CD4^+^ T cells with the inset showing hnRNP L expression in WT and KO CD4^+^ T cells before stimulation. T cells from WT and KO mice were stimulated with IL-4, anti-IFNγ, and IL-2 to induce Th2. **(E)** Representative flow plot of GATA3 ^+^ CD25^+^ T cells three days post stimulation, gated on CD4^+^ T cells **(F)** the percentage of GATA3^+^ CD4^+^ T cells over several experiments, and **(G)**, and a histogram of hnRNP L expression in WT (filled histogram) and KO (dotted line) GATA3^+^ CD4^+^ T cells with the inset showing hnRNP L expression in WT and KO CD4^+^ T cells before stimulation. T cells from WT and KO mice were stimulated with TGF-β, IL-6, anti-IFNγ, anti-IL4, and IL-2 to induce Th17. **(H)** Representative flow plot of RORγT^+^ CD25^+^ skewed T cells three days post stimulation, gated on CD4^+^ T cells **(I)** the percentage of RORγT^+^ CD4^+^ T cells over several experiments **(J)**, and a histogram of hnRNP L expression in WT (filled histogram) and KO (dotted line) RORγT^+^ CD4^+^ T cells with the inset showing hnRNP L expression in WT and KO CD4^+^ T cells before stimulation in top left corner. T cells from WT and KO mice were stimulated with TGF-β, IL-2, anti-IFNγ, and anti-IL4 to induce Treg cells. **(K)** Representative flow plot of Foxp3^+^ CD25^+^ T cells three days post stimulation, gated on CD4^+^ T cells, **(L)** the percentage of Foxp3+ Tregs **(M)**, and a histogram of hnRNP L expression in WT (filled histogram) and KO (dotted line) Foxp3^+^ CD4^+^ T cells with the inset showing hnRNP L expression in WT and KO CD4^+^ T cells before stimulation. Data in **(C, F, I, L)** represent the combination of three independent experiments (n = 1-2 mice per group). Unpaired two-tail Student T-test was performed *p < 0.05, ****p < 0.0001.

To determine whether loss of hnRNP L affected early T cell activation, a T cell activation assay was performed. Total CD4^+^ and CD8^+^ T cells were purified from the spleens of 3 WT strains (CD4Cre^+^ hnrnpl^wt/wt^, CD4Cre^-^ hnrnpl^wt/wt^, CD4Cre^-^ hnrnpfl^fl/fl^) and the KO strain (CD4Cre^+^ hnrnpl^fl/fl^). Purified T cells were activated on anti-CD3 mAb coated plates in the presence of soluble anti-CD28 mAb, and the cells were examined at various time points for the activation markers, CD69 (**Figure 2A**), and for phosphorylation of S6 ribosomal protein (pS6), which is downstream of the PI3K/Akt/mTOR pathway. Upon TCR stimulation there were no differences in pS6 expression between the CD4^+^ WT and KO T cells at 6 and 24 hours post-stimulation (**Figure 2A**). Similar results were seen when gating on the CD8^+^ T cells (**Supplementary Figure 2A**). These results suggest that early activation steps in hnRNP L KO CD4^+^ T cells proceed normally.

### HnRNP L is required for Th subset differentiation

To investigate the effects of loss of hnRNP L on CD4^+^ T cell effector fate, T cells from WT and KO spleens were cultured *in vitro* to induce Th1, Th2, Th17, or Tregs. T cells from WT and KO mice were stimulated under various conditions, as described in the methods. After 3 days of culture, the cells were examined for expression of the canonical transcription factors characteristic of each Th cells subset. Representative flow plots for each culture condition are shown (**Figures 2B, E H, K**) along with quantitation of the proportion of differentiated cells across several experiments (**Figures 2C, F, I, L**). HnRNP L deficient T cells had a significant reduction in the percentages of Th1 (**Figure 2C**), Th2 (**Figure 2F**), and Tregs (**Figure 2L**), induced by the presence of *in vitro* skewing cytokines. There was no significant difference in the percentage of RORγT-expressing Th17 cells (**Figure 2I**), although these were highly variable. However, in all cases, it can be seen from the flow plots that there was an overall reduction in the number of viable cells at the end of the culture of the KO cells. We examined the expression pattern of hnRNP L before and after stimulation. In all cases, the KO cells started the culture with a majority of hnRNP L-deleted cells (inset in **Figures 2D, G, J, M**). However, by day 3, the cells that differentiated into the different Th cell subsets, as determined by canonical transcription factor expression, were now all expressing significant levels of hnRNP L (**Figures 2D, G, J, M**). These results suggest that only those cells that failed to delete hnRNP L were able to differentiate into Th cell subsets and suggested that the KO T cells had died.

### HnRNP L is required for T-dependent germinal center formation

The previous experiments were all performed *in vitro* using antibody-mediated stimulation in the absence of antigen presenting cells (APCs), and it was possible that the KO T cells were missing additional signals from APCs. We examined the differentiation of Tfh cells using an *in vivo* model of NP-KLH immunization (71). In this model, WT (CD4Cre^+^ hnrnpl^wt/wt^, and CD4Cre^-^ hnrnpfl^fl/fl^) or KO mice were immunized with NP-KLH, and after 10 days, the spleens were examined for the presence of Tfh cells, NP-specific B cells, and GC formation.

**Figure 3:**
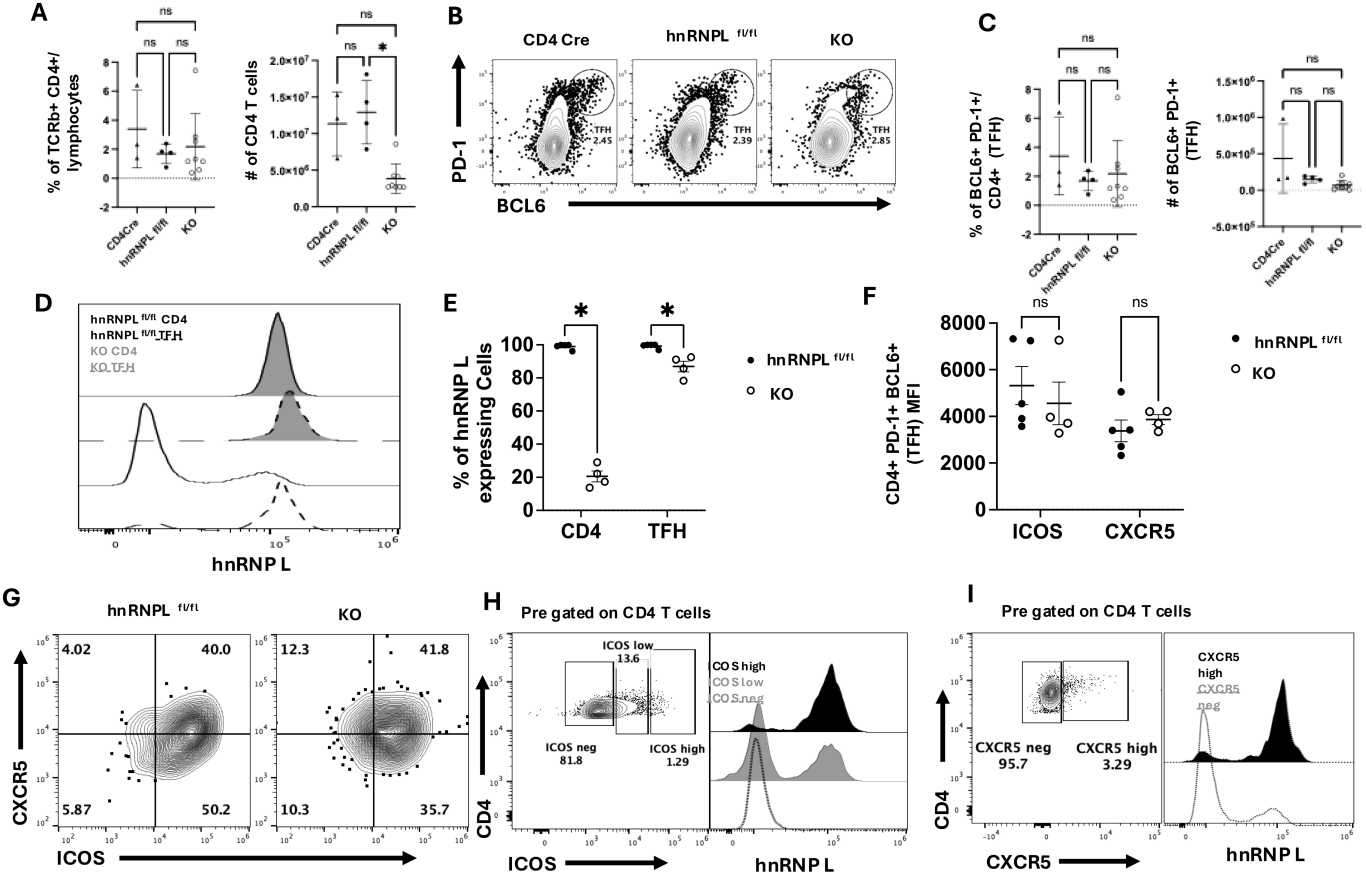
HnRNP L is required for optimal Tfh survival and T-dependent germinal center formation. WT (CD4Cre^+^ hnrnpl^wt/wt^, CD4Cre^-^ hnrnpfl^fl/fl^) or KO (CD4Cre^+^ hnrnpfl^fl/fl^) mice were immunized with 100 μg of NP-KLH, spleens were harvested 10 days post-immunization and analyzed for the presence of Tfh cells. **(A)** The frequency and the number of total CD4^+^ T cells in the spleen of immunized WT and KO mice. **(B)** Representative flow plot of Tfh profile gated on CD4^+^ T cells. Tfh cells were defined as PD-1^+^ BCL6^+^ and **(C)** the frequency (left panel) and the number (right panel) of Tfh cells in the spleen of WT and KO immunized mice. **(D)** Representative flow plot of hnRNP L expression in WT (filled histogram) vs. KO (empty histogram) Tfh (dotted lines) CD4^+^ T cells and non-Tfh (solid lines) CD4^+^ T cells. **(E)** The quantification of the % of hnRNP L expressing cells total CD4^+^ and gated Tfh T cells. **(F)** The MFI of ICOS and CXCR5 expression in Tfh from WT and KO mice. **(G)** Representative flow plot of CXCR5 vs. ICOS expression on pre-gated Tfh cells from immunized WT and KO mice. **(H)** Representative flow plot of ICOS expression (left panel) in CD4^+^ T cells from immunized KO mice and hnRNP L expression (right panel) within the ICOS^high^ (black histogram), ICOS^low^ (grey histogram) and ICOS^neg^ (empty histogram) CD4^+^ T cell populations. **(I)** Representative flow plot of CXCR5 expression (left panel) in CD4^+^ T cells from immunized KO mice and hnRNP L expression (right panel) within the CXCR5^high^ (black histogram) and CXCR5^neg^ (empty histogram) CD4^+^ T cell populations. Results represent the mean+/- SD of 3 independent experiments with 2-3 mice per group. Unpaired two-tail Student T-test and a two-way ANOVA analysis with Tukey posttest were performed *p < 0.05. ns, not significant.

We observed a reduction in the total number of T cells in the spleen of immunized KO mice (**Figure 3A**). Tfh cells were identified based on PD-1 and BCL6 expression (**Figure 3B**), and there were no significant differences in the percentages or number of Tfh cells between WT and KO immunized mice (**Figure 3B**). There were no differences in the percentage of number of CD4^+^ PD-1^+^ BCL 6^-^ T cells (**Figure 3C**). When we examined the Tfh cells for hnRNP L expression, we saw that all the Tfh cells in the KO mice expressed wild type levels of hnRNP L (**Figure 3D**). In contrast, the non-Tfh CD4^+^ T cells in the same mice were still hnRNP L deficient (**Figures 3D, E**). Tfh cells are known to express ICOS and CXCR5, and we examined the expression of these markers in WT and KO Tfh cells. Expression levels of ICOS and CXCR5 were similar in gated Tfh cells from WT and KO mice (**Figures 3F, G**). When gating on ICOS^neg^, ICOS^low^, and ICOS^hi^ CD4^+^ T cells we noted that ICOS^neg^ cells expressed KO levels of hnRNP L whereas ICOS^hi^ cells expressed wild type levels of hnRNP L, with ICOS^low^ cells contained cells with or without hnRNP L deletion (**Figure 3H**). We observed a similar phenomenon when gating on CXCR5^neg^ and CXCR5^pos^ CD4^+^ T cells (**Figure 3I**). Thus, we conclude that the *in vivo* environment does not correct the differentiation defect in hnRNP L KO cells and that only those cells that have retained hnRNP L expression can differentiate into Tfh cells.

**Figure 4:**
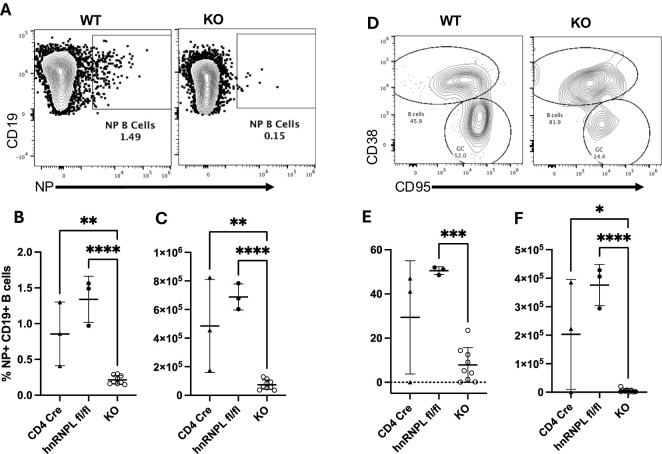
HnRNP L is required for germinal center (GC) formation. WT (CD4Cre^+^ hnrnpl^wt/wt^, CD4Cre^-^ hnrnpfl^fl/fl^) or KO mice were immunized with 100ug of NP-KLH, the spleen was harvested 10 days post-immunization and analyzed for the presence of NP-specific B cells and GC formation. **(A)** Representative flow plot of the identification of NP^+^ B cells in WT and KO immunized mice. **(B)** The percentage and **(C)** absolute number of NP^+^ B cells in the spleen of WT and KO immunized mice. **(D)** NP specific B cells were stained with CD38 and CD95 to identify GC (CD38^-^ CD95^+^) and non-GC (CD38^+^ CD95^-^) B cells. Representative flow plot of CD38/CD95 staining on NP-specific B cells in WT and KO immunized mice. **(E)** The percentage, and **(F)** the absolute number of NP-specific GC B cells in WT and KO immunized mice. Results represent the mean+/- SD of 3 independent experiments with 2-3 mice per group. Unpaired two tail T-test was performed, *p<0.05, **p < 0.01, ***p<0.001 ****p < 0.0001.

We examined the spleens of the same immunized mice for the presence of NP-specific B cells ten days post-immunization by enumerating NIP^+^ CD19^+^ B cells (**Figure 4A**). KO mice had a reduced frequency (**Figure 4B**) and number (**Figure 4C**) of NP-specific B cells. The NP-specific population was further analyzed for the presence of CD95^+^ CD38^-^ GC B cells (**Figure 4D**). There was a marked reduction in the proportion (**Figure 4E**) and number (**Figure 4F**) of NP-specific GC B cells in the KO mice. Thus, even though we could detect the presence of Tfh in KO mice, these cells were unable to support the generation of antigen-specific B cells or differentiation into GC B cells.

### HnRNP L is required for T cell proliferation and survival following activation

**Figure 5:**
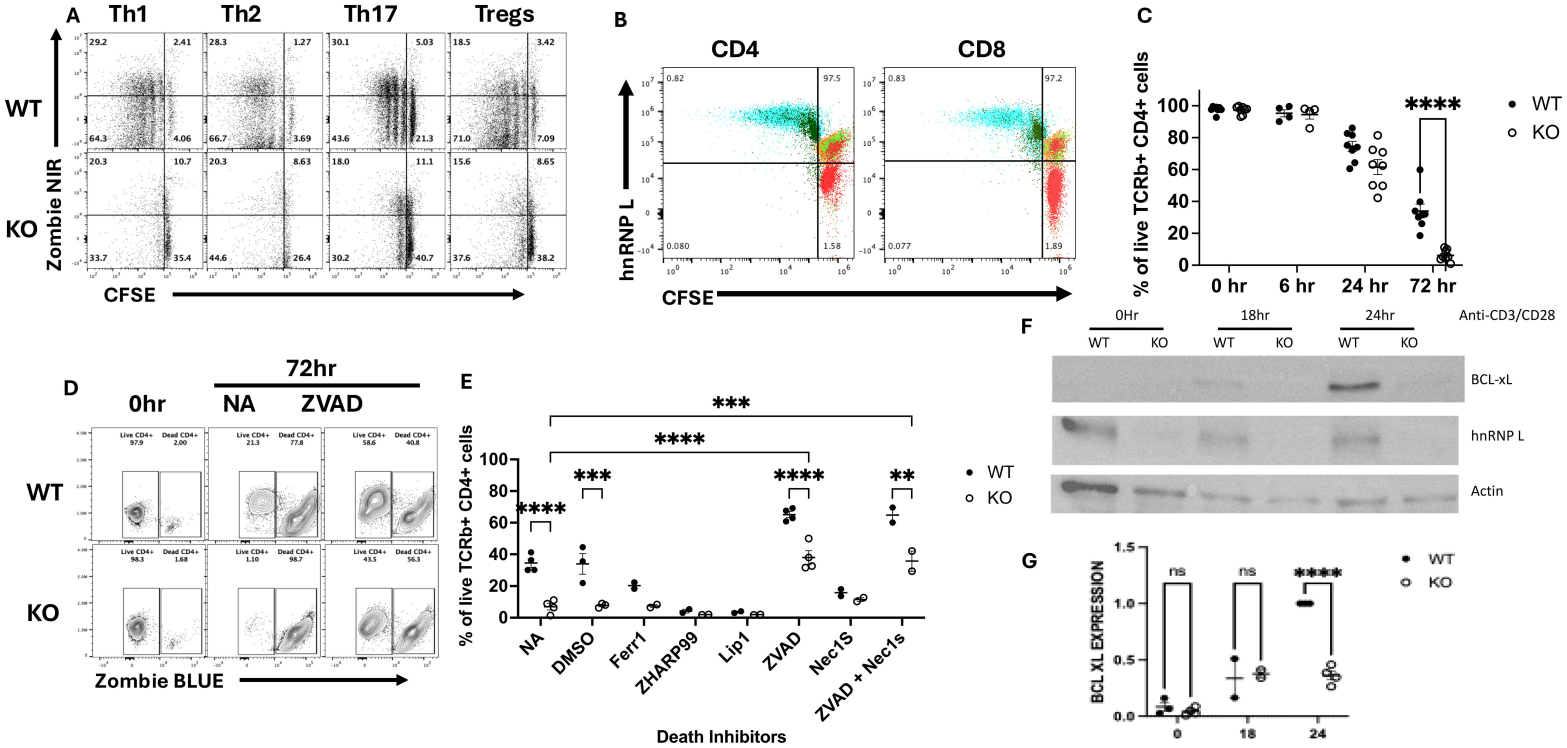
HnRNP L is required for T cell proliferation and survival following activation. **(A, B)** WT or KO T cells were isolated using a pan T cell kit, CFSE labeled and stimulated under Th subset cell skewing conditions for 72 hours as described in the legend to **Figure 3**. **(A)** Representative flow plot of CFSE vs Zombie NIR in WT (upper panels) and KO (lower panels) CD4^+^ T cells stimulated for 72 hours with the indicated skewing conditions. **(B)** Flow cytometry analysis of CFSE dilution and hnRNP L expression in CD4 (left) and CD8 (right) cells before and after stimulation. Flow cytometric analysis of CFSE dilution was performed on cells at T=0 and 3 days after Th1 stimulation on gated CD4 and CD8 T cells. The red cells are KO CD4 or CD8 T cells prior to stimulation. The green and orange cells are WT (green) and Het (orange) CD4 or CD8 T cells prior to stimulation. The blue and dark green cells are WT (blue) and KO (dark green) CD4 or CD8 cells 3 days after Th1 stimulation. **(C)** 2-2.5x10^5^ T cells were plated in a 96-well plate with 3.0 µg/ml plate-bound anti-CD3 in the presence of soluble 3.0 µg/ml anti-CD28. The cells were harvested at the indicated times points and % of live cells, (gated on CD4^+^ T cells) is presented for both WT and KO cells. **(D, E)** 2-2.5x10^5^ WT or KO T cells were incubated with death pathway inhibitors zVAD (1000X), ZHAR-99 (500X), Lip1 (500 X), Fer-1 (500X) and DMSO for 1 hour followed activation with 3.0 µg/ml plate-bound anti-CD3 and soluble 3.0 µg/ml anti-CD28. The cells were harvested after 6, 24 and 72 hrs post stimulation. **(D)** Representative flow plot of cell viability at 72 hours in the absence (NA) or presence of zVAD. **(E)** The percentage of live cells detected at 72 hours in the absence or presence of the indicated death inhibitors. NA - No addition; DMSO - vehicle control **(F)** Western blot for BCL-X_L_, hnRNP L and actin expression at 0, 18- and 24-hrs post-stimulation. **(G)** The quantification of BCL-X_L_ expression in three independent experiments. The values presented represent the relative amount of actin-normalized BCL-X_L_ expression compared to the maximal amount seen in WT T cells at 24 hrs. Unpaired two tail T-test **(B)** and two-way ANOVA analysis with Tukey posttest **(C, E, G)** were performed, **p < 0.01, ***p < 0.001, ****p<0.0001. ns, not significant.

Our findings above indicate that hnRNP L-deficient T cells exhibit impaired activation and survival in both *in vitro* and *in vivo* experimental models. To further elucidate the underlying mechanisms, we conducted a detailed analysis of T cell proliferation and cell death kinetics following *in vitro* stimulation. Flow cytometric analysis of CFSE-labeled WT T cells revealed significant proliferation at 72 hours post-stimulation with anti-CD3/CD28 mAbs, accompanied by a small subset of proliferated cells positive for Zombie NIR, indicating compromised viability (**Figure 5A**, upper panel). In contrast, most KO T cells were Zombie NIR positive after 72 hrs stimulation and very few showed evidence of CFSE dilution (**Figure 5A**, lower panel). To determine whether the few cells that had diluted CFSE were in fact hnRNP L KO we co-stained the cells with hnRNP L (**Figure 5B**). The KO cells at T=0 mostly do not express hnRNP L but there is a small population that expresses WT levels of hnRNP L (see small red population that overlaps with the WT and Het cells). After activation the expression level of hnRNP L goes up and is similar in the WT and KO CD4^+^ and CD8+ T cells. The CFSE is also diluted in both activated populations, compared to the T=0 cells (**Figure 5B**, blue and dark green populations). Thus, we can conclude that only those cells that had failed to delete hnRNP L were able to divide. Temporal assessment of cell death demonstrated a marked increase in death after 72 hours of TCR stimulation (**Figure 5C**). A similar pattern was seen in CD8^+^ T cells (**Supplementary Figure 2B**) Upon TCR engagement, activated T cells typically undergo activation-induced cell death (AICD), a homeostatic process that limits excessive immune responses (24). AICD is predominantly mediated by the Fas/FasL (CD95/CD178) signaling axis, which initiates apoptotic cascades. To determine the method of cell death we pretreated the cells with various death inhibitors prior to stimulation and the apoptotic nature of AICD was confirmed by the protective effect of the pan-caspase inhibitor z-VAD-FMK, in contrast to the inefficacy of ferroptosis (Ferrostatin-1, Liproxstatin-1) or necroptosis (ZHARP-99, Necrostatin-1s) inhibitors (**Figures 5D, E**). The cell death observed in KO T cells appeared to be caspase-dependent, as evidenced by z-VAD-FMK-mediated protection (**Figures 5D, E**).

Given the apparent AICD-associated apoptosis in hnRNP L-deficient T cells, we investigated potential defects in the induction of anti-apoptotic molecules, focusing on BCL-X_L_, a well-characterized protein upregulated following activation of naïve CD4^+^ cells (25). Western blot analysis revealed detectable BCL-X_L_ expression beginning at 18 hours post-activation of naïve CD4^+^ cells, with maximal levels observed at 24 hours. Strikingly, hnRNP L-deficient T cells displayed a marked reduction in BCL-X_L_ expression at the 24-hour time point (**Figure 5F**). Control immunoblots confirmed hnRNP L deletion and equal protein loading using anti-hnRNP L and anti-β-actin antibodies, respectively (**Figure 5F**). These findings were consistently reproducible across several independent experiments (**Figure 5G**). Collectively, these data suggest that the absence of hnRNP L predisposes T cells to AICD-associated apoptosis, potentially due to impaired induction of the anti-apoptotic factor BCL-X_L_. This molecular mechanism may, at least in part, explain the observed activation and survival defects in hnRNP L-deficient T cells.

## Discussion

The experiments presented here demonstrate the importance of hnRNP L in T cell proliferation, survival, and death. Using CD4^cre^ x Hnrnpl^fl/fl^ mice, we observed that hnRNP L knockout T cells develop normally in the thymus and migrate to the peripheral lymphoid organs. There were reduced numbers and percentages of total T cells in the spleen and reduced numbers in peripheral lymph nodes. Early activation via TCR signaling appeared normal, as determined by measuring pS6 and upregulation of CD69. We showed that hnRNP L deficient T cells were unable to differentiate into Th1, Th2, Th17, and Tregs *in vitro*, as the only cells that differentiated were those that failed to delete hnRNP L. Using the NP-KLH model, a T cell-dependent immunization model, we observed no change in the number or percentage of Tfh and CD4^+^ T cells in CD4^cre^ x *Hnrnpl^fl/fl^* mice. However, as in the *in vitro* system, all the observed Tfh cells in the KO mice had failed to delete hnRNP L. The Tfh in the CD4^cre^ x *Hnrnpl^fl/fl^* mice did not support the formation of GC in the spleen or the expansion of NP-specific B cells. Finally, we observed that activated hnRNP L KO T cells failed to proliferate following activation and that they underwent apoptosis. The apoptosis phenotype appeared to be related to a failure to upregulate the anti-apoptotic protein BCL-X_L_ in KO T cells.

Unlike in the Lck^cre^ x *Hnrnpl^fl/fl^* mouse model, where thymic development was perturbed and only T cells that failed to delete hnRNP L migrated to the periphery (17) the CD4^cre^ x *Hnrnpl^fl/fl^* mouse model had normal thymic development and the mature T cells in the periphery did not express hnRNP L, though the number of T cells was reduced, making this model a useful tool to study the role of hnRNP L in mature T cell function. The lack of a thymic phenotype appeared to be due to persistent hnRNP L expression at DP and SP stages of thymic development, possibly due to the protein half-life (data not shown). However, we did note a decrease in CD5 expression in KO SP cells suggesting that cells receiving lower TCR signals were preferentially selected in KO animals (22, 23). While most peripheral T cells in this model exhibited hnRNP L deletion, small populations of CD4^+^ and CD8^+^ T cells retained hnRNP L expression, which became relevant in subsequent assays. The CD4^cre^ is thought to have high deletion efficiency (29), with few reports of leakiness. This study demonstrates that deletion of molecules that play an important role in the overall survival of cells may not always be complete, even with strong Cre-Lox models.

Initial characterization of the function of hnRNP L KO T cells revealed that early activation signals via the TCR pathway occurred normally, for at least the first 6-24 hours. Both WT and KO T cells initially up-regulated activation markers like CD69 and activated the PI3K/Akt/mTOR pathway as evidenced by pS6 expression. However, longer periods of activation resulted in death of the KO T cells with only T cells that escaped hnRNP L deletion surviving beyond three days. We performed assays to induce Th subset differentiation both *in vitro* and *in vivo*, but hnRNP L-deficient T cells were functionally deficient and more prone to die, and the only cells that successfully underwent Th cell differentiation had escaped hnRNP L deletion. Thus, it appears that hnRNP L plays a fundamental role in many important cellular functions that, if dysregulated, could induce cell death.

Interestingly the *in vivo* immunization experiments revealed that although there appeared to be induction of hnRNP L-positive Tfh cells there was a marked defect in NP-specific B cell expansion and GC formation. As yet, this is unexplained, but it may be related to the low initial frequency of specific Tfh cells which could have resulted in a delay in the interaction between NP specific B cells and KLH-specific T cells. We did examine the B cell compartment in hnRNP L KO mice and we did not detect any gross abnormalities in the number or phenotype of B cell subsets in the spleen (data not shown).

To determine which cell death pathways could be involved in the death of hnRNP L KO T cell we performed a screen using inhibitors of each of the major cell death pathways. Necroptosis was blocked by Zharp-99, a RIPK3 inhibitor (26) or Necrostatin-1s, a RIPK1 inhibitor (26). Ferroptosis was blocked by Lip-1 or Ferr-1 (27), caspases-mediated apoptosis and pyroptosis were blocked by zVAD, a pan-caspase inhibitor (28). Only the pan-caspase inhibitor zVAD was able to reverse cell death, whereas the inhibitors of ferroptosis and necroptosis had no impact on cell death and, in some cases, appeared to enhance cell death. While pan-caspase inhibition via zVAD significantly attenuated cell death in both wild-type (WT) and hnRNP L-deficient T cells following TCR engagement, complete prevention was not achieved. This observation warrants several considerations. Firstly, the incomplete protection may be attributed to the gradual degradation of zVAD during our extended culture periods. Alternatively, this phenomenon could be indicative of caspase-independent cell death (CICD) mechanisms. CICD typically occurs in response to apoptotic stimuli when caspase activity is pharmacologically inhibited and proceeds downstream of mitochondrial outer membrane permeabilization (MOMP) (29). It is also crucial to consider that the enhanced susceptibility to cell death observed in hnRNP L-deficient T cells may result from a complex interplay of multiple cell death pathways, potentially exacerbated by impaired activation and survival signaling. Future studies employing a combination of genetic and pharmacological approaches will be instrumental in dissecting these intricate mechanisms and their potential interconnections in the context of hnRNP L deficiency.

Other groups have shown, using different Cre models, that loss of hnRNP L causes an increase in ROS that is associated with mitochondrial dysfunction (18, 20). HnRNP L regulates NFAT (18), which regulates oxidative stress in the mitochondria (30). HnRNP L has been shown to upregulate the expression of the death receptors CD95/Fas and Trail R2 (20). DNA damage may occur when cells divide and when DNA damage occurs, p53 expression increases. HnRNP L-deficient hematopoietic stem cells (HSC) have higher expression of p53 and p53 effectors (20), but the deletion of p53 did not restore HSC survival (20). HnRNP L can also associate with BCL-2 mRNA, which also plays a role in cell death (31). BCL-2 is an anti-apoptotic protein that can inhibit the mitochondrial outer membrane permeabilization thus can limit the activation of caspases 3, 6, and 9 (32),. The CA repeats of BCL-2 mRNA are known to contribute to the decay of BCL-2 mRNA, but increasing or decreasing the expression of hnRNP L did not change the rate of BCL-2 mRNA degradation (31, 33). BCL-X_L_ is a member of the BCL-2 family, and like BCL-2, BCL-X_L_ is also anti-apoptotic protein that can inhibit apoptosis (32).

We measured BCL-X_L_ expression following T cell activation and showed that hnRNP L-deficient T cells failed to induce expression of the anti-apoptotic molecule BCL-X_L_ at 24 hours. Thus, it is possible that hnRNP L-deficient T cells are dying because of the failure to induce pro-survival factors, such as BCL-X_L._ One group showed that increased expression of BCL-X_L_ in human T cells conferred resistance to CD95-induced cell death (34). BCL-X_L_ was found to act independently and downstream of caspase 8 (35), which is needed by T cells to maintain homeostasis and activation (28). BCL-X_L_ expression is controlled by alternative splicing (36). Two isoforms of BCL-X have been identified, a long isoform BCL-X_L_ which is anti-apoptotic, and a short form BCL-X_S_ which is pro-apoptotic. BCL-X_L_ acts to inhibit Bax and Bak under conditions of cellular stress such as oxidative stress, thus preserving mitochondrial membrane integrity. BCL-X_S_ inhibits the action of BCL-X_L_, and the ratio between these two isoforms plays an important role in determining cell death. Several RBP have been identified as playing a role in BCL-X_L_ splicing (36), including SRSF1 and several hnRNPs. A recent study demonstrated that hnRNP L could influence splicing in a mini gene derived from BCL-X, possibly by inhibiting SRSF1 action (37), suggesting that the lack of hnRNP L in T cells could influence the relative levels of BCL-X_S_ and BCL-X_L_. Further studies are required to determine whether hnRNP L is directly involved in the alternative splicing of BCL-X isoforms.

Thus, it is possible that hnRNP L is involved in the alternative splicing of multiple proteins involved in cell death, resulting in enhanced survival. Deletion of hnRNP L in peripheral T cells results in an inability of T cells to survive, notably after activation when multiple changes in metabolism and protein expression are required. A recent paper examined a B cell-specific deletion of hnRNP L (38), and this was associated with proliferation defects and increased apoptosis following activation. This study identified defects in isotype switching and pathways important for cell survival such as Myc (38). Our studies suggest that hnRNP L plays a critical pro-survival role in activated T cells and that alternative splicing of factors that prevent apoptosis may be an important mechanism by which this is achieved.

## Data Availability

The original contributions presented in the study are included in the article/**Supplementary Material**. Further inquiries can be directed to the corresponding author.
