# Supplementary material for: HnRNP L is essential for peripheral T cell proliferation and survival
Source: Front Immunol. 2025 Apr 10;16:1543145. doi: 10.3389/fimmu.2025.1543145 (PMC12018431; doi:10.3389/fimmu.2025.1543145)
Supplement: Supplementary file 1 [file Image1.pdf]

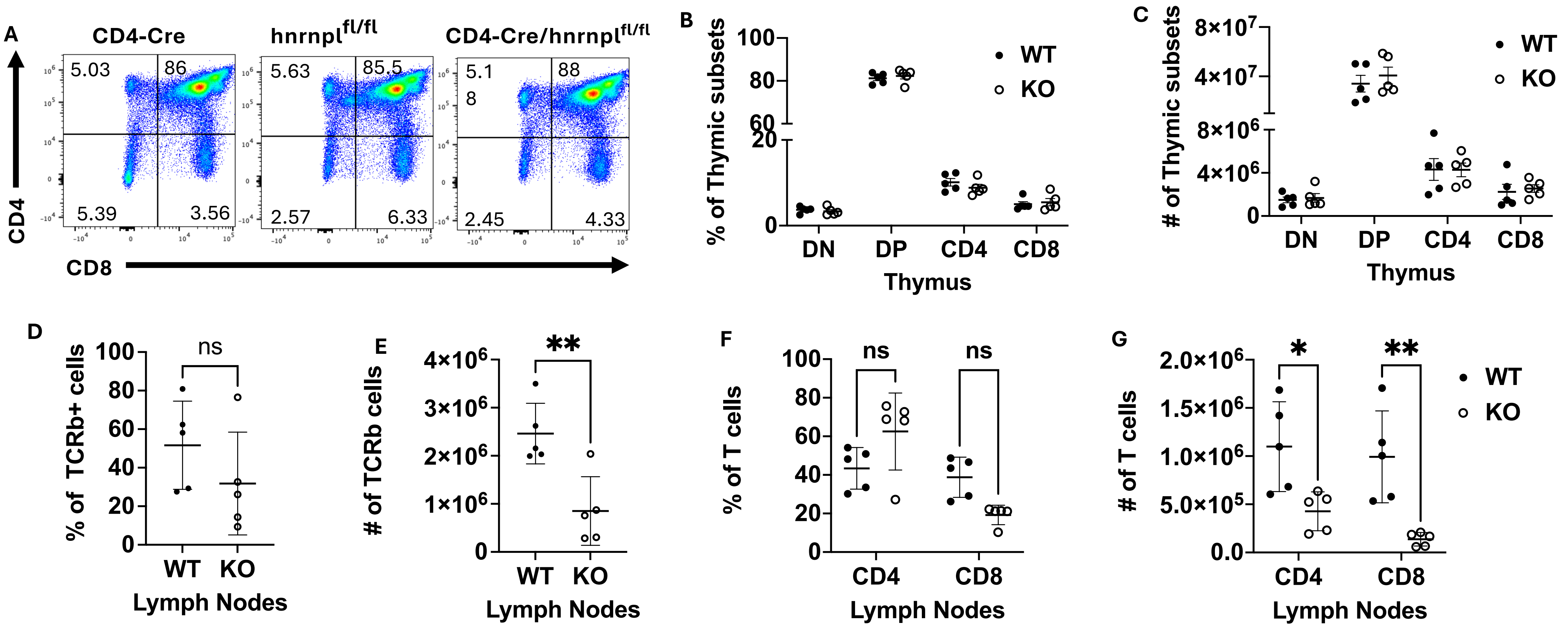

Supplemental Figure 1: Assessment of thymus development and LN populations in hnRNPL KO mice. Thymocytes were isolated from WT (CD4Cre^{-} hnrnpfl$^{fl/fl}$) and KO mice to assess T cell development. A representative flow plot of thymic populations **(A)** pre-gated on live thymocytes. **(B)** The frequency and the **(C)** number of the indicated thymocyte populations. The frequency **(D)** and number **(E)** of TCRβ^{+} and frequency **(F)** and number **(G)** CD4^{+} and CD8^{+} T cells in the lymph nodes**.** *p<0.05, **p<0.01
