# Supplementary material for: HnRNP L is essential for peripheral T cell proliferation and survival
Source: Front Immunol. 2025 Apr 10;16:1543145. doi: 10.3389/fimmu.2025.1543145 (PMC12018431; doi:10.3389/fimmu.2025.1543145)
Supplement: Supplementary file 2 [file Image2.pdf]

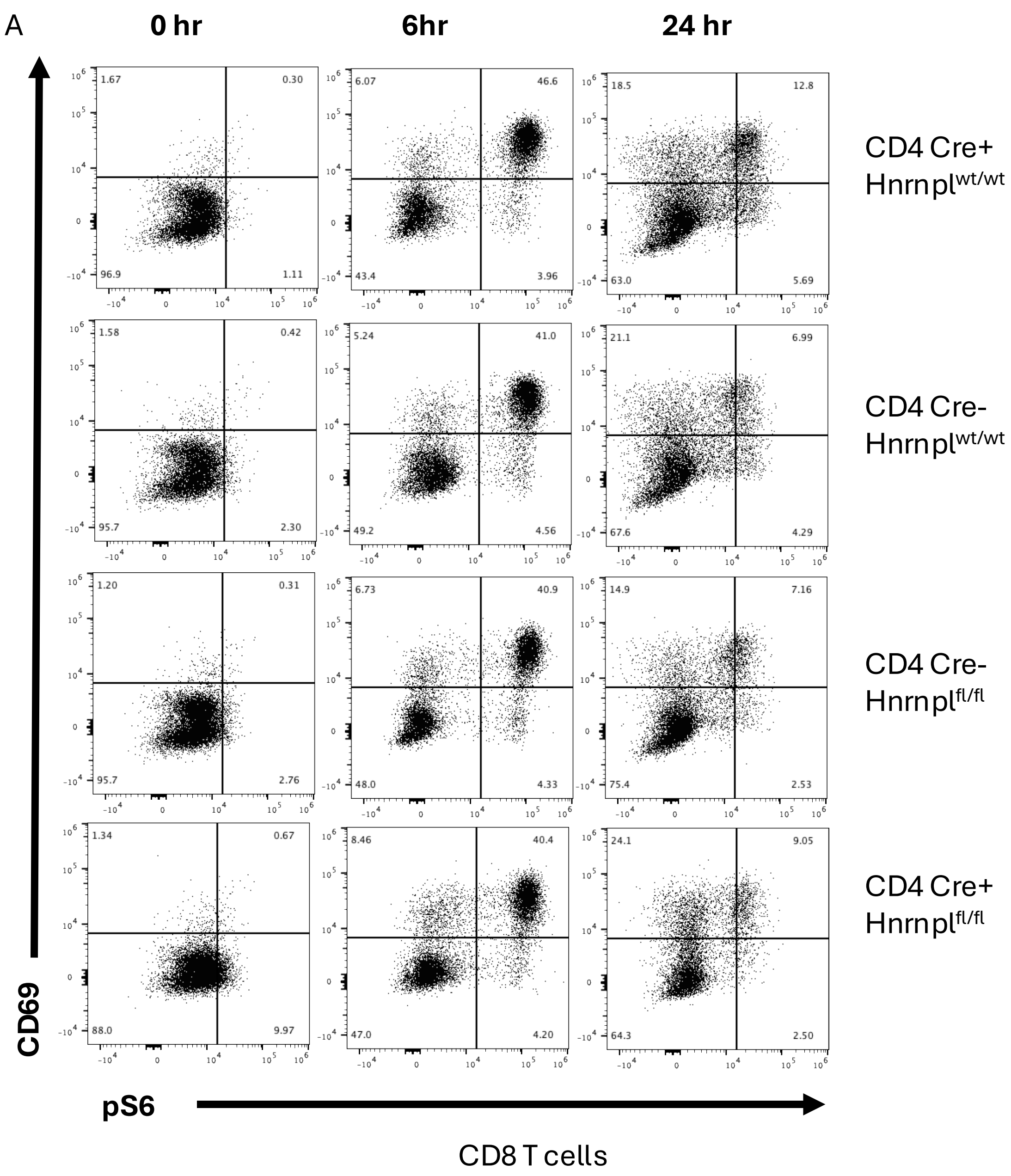

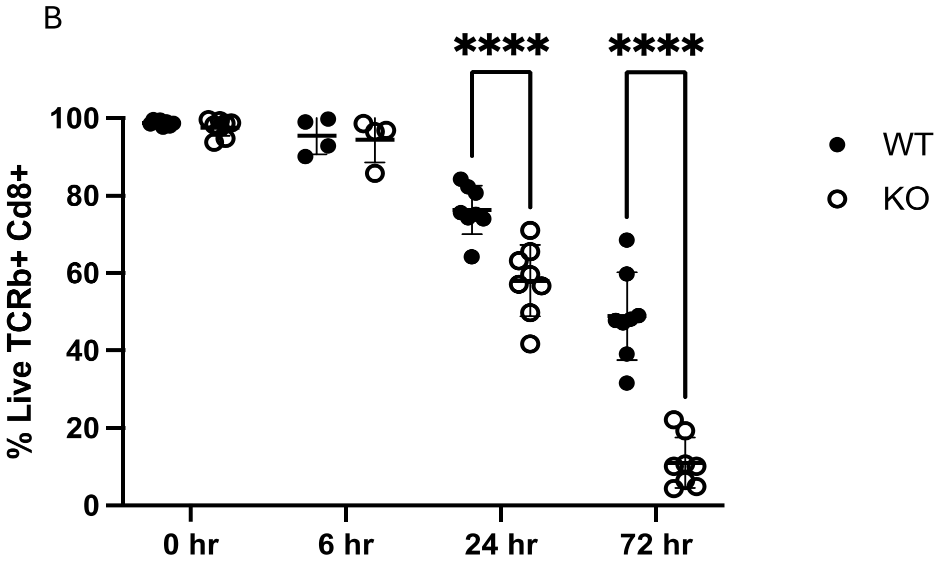

Supplemental Figure 2. Early activation is normal in KO CD8 T cells, but significant cell death occurs 72 hours post activation. T cells from WT (CD4Cre^{+} hnrnpl$^{wt/wt}$, CD4Cre^{-} hnrnpl$^{wt/wt}$, CD4Cre^{-} hnrnpfl$^{fl/fl}$) and KO (CD4Cre^{+} hnrnpfl$^{fl/fl}$) mice were isolated using a negative selection pan T cell isolation kit. 2-2.5x10^{5} T cells were plated in a 96-well plate with 3.0 µg/ml plate-bound anti-CD3 in the presence of soluble 3.0 µg/ml anti-CD28. The cells were harvested after various time points. **(A)** Representative flow plot of stimulated CD8^{+} T cell expression of CD69 vs pS6 at the indicated time points. **(B)** 2-2.5x10^{5} T cells were plated in a 96-well plate with 3.0 µg/ml plate-bound anti-CD3 in the presence of soluble 3.0 µg/ml anti-CD28. The cells were harvested at the indicated times points and % of live cells, (gated on CD8^{+} T cells) is presented for both WT and KO cells. ****$p<0.0001$
